# Critical factors in cut-out complication after gamma nail treatment of proximal femoral fractures

**DOI:** 10.1186/1471-2474-14-1

**Published:** 2013-01-02

**Authors:** Alicja J Bojan, Claudia Beimel, Gilbert Taglang, David Collin, Carl Ekholm, Anders Jönsson

**Affiliations:** 1 Department of Orthopaedics, Institute of Clinical Sciences at Sahlgrenska Academy, Gothenburg University, Gothenburg, Sweden; 2 Stryker Osteosynthesis, Schönkirchen, Germany; 3 Trauma Unit University Hospital of Strasbourg, Strasbourg, France

## Abstract

**Background:**

The most common mechanical failure in the internal fixation of trochanteric hip fractures is the cut-out of the sliding screw through the femoral head. Several factors that influence this complication have been suggested, but there is no consensus as to the relative importance of each factor.

The purpose of this study was to analyse the cut-out complication with respect to the following variables: patients’ age, fracture type, fracture reduction, implant positioning and implant design.

**Methods:**

3066 consecutive patients were treated for trochanteric fractures with Gamma Nails between 1990 and 2002 at the Centre de Traumatologie et de l`Orthopedie (CTO), Strasbourg, France. Cut-out complications were identified by reviewing all available case notes and radiographs. Subsequently, the data were analysed by a single reviewer (AJB) with focus on the studied factors.

**Results:**

Seventy-one cut-out complications were found (2.3%) of the 3066 trochanteric fractures. Cut-out failure associated with avascular head necrosis, pathologic fracture, deep infection or secondary to prior failure of other implants were excluded from the study (14 cases). The remaining 57 cases (1.85 %, median age 82.6, 79% females) were believed to have a biomechanical explanation for the cut-out failure. 41 patients had a basicervical or complex fracture type. A majority of cut-outs (43 hips, 75%) had a combination of the critical factors studied; non-anatomical reduction, non-optimal lag screw position and the characteristic fracture pattern found.

**Conclusions:**

The primary cut-out rate of 1.85% was low compared with the literature. A typical cut-out complication in our study is represented by an unstable fracture involving the trochanteric and cervical regions or the combination of both, non-anatomical reduction and non-optimal screw position. Surgeons confronted with proximal femoral fractures should carefully scrutinize preoperative radiographs to assess the primary fracture geometry and fracture classification. To reduce the risk of a cut-out it is important to achieve both anatomical reduction and optimal lag screw position as these are the only two factors that can be controlled by the surgeon.

## Background

The treatment of proximal femoral fractures continues to be less than optimal due to a moderate complication rate. The most commonly reported complication in the internal fixation is the cut-out defined as “the collapse of the neck-shaft angle into varus, leading to extrusion of the screw from the femoral head“ [1]. Several studies have shown that the incidence of cut-out for different compression hip screws and intramedullary nails ranges from 0 to 16.5% [2-5], and in older studies [6,7] even up to 17.5 - 20%.

This complication is a multifactorial event affected by a number of variables including patient’ age, bone quality, fracture pattern, quality of reduction, lag screw positioning in the femoral head, implant design and the choice of CCD-nail angle [1,8]. These factors have also been frequently discussed in the literature, however there has been no clear consensus either to their interrelationships or to the relative importance of each [1].

The aim of the present study was to analyse cut-out complication in patients treated with Gamma Nails in order to obtain a clearer understanding of interrelations of critical factors contributing to the mechanism of cut-out. The critical factors assessed were patients’ age, type of the fracture, quality of reduction, positioning of the lag screw, neck-shaft angle of the implant and implant design. It is hoped that the findings may better guide the surgeon in the prevention of this complication.

## Methods

The present study is a continuation of the previously published work by Bojan et al. [9]. All patients with trochanteric, subtrochanteric or combined trochantero-diaphyseal fractures entering the Centre de Traumatologie et de l`Orthopedie (CTO), Strasbourg, France between the 1^st^ of January 1990 to the 31^st^ of December 2002 were treated with Gamma nails (Standard Gamma Nail, Trochanteric Gamma Nail, Long Gamma Nail).

The patients were treated as surgical emergencies and the procedures were performed both by doctors under training and by senior surgeons. All surgeons were trained for the procedure. The patients were operated on a traction table in a supine position, general and spinal anaesthesia being equally common. Image intensifier was used. Additional fixation methods such as screws, cerclage wires and bone grafting were used when considered appropriate. Full weight bearing was allowed immediately post-operatively, except when fixation was assessed as being insufficiently stable. Radiological examinations were performed pre-operatively, post-operatively within 24 hours after surgery and at follow-up when indicated.

Parameters such as fracture type according to the AO/ASIF system and position of the lag screw in the femoral head were assessed for the whole study group.

The cut-out complications were detected with the help of surgical reports, radiographs and follow-up visit notes. Patients were routinely scheduled for follow-up visits between 3 and 6 months post-operatively. Exceptions were made for patients hospitalised at other institutions. The analogue radiographs of all cut-outs were digitalised with help of a Fujifilm FinePix S1Pro camera. Reduction of the fracture was assessed on immediate post-operative radiographs. For the reduction to be considered anatomical, there had to be a normal alignment (meaning 160° [10]) on the antero-posterior (A-P) radiograph, less than 20° of angulation on the lateral radiograph, and no more than four millimetres of displacement of any fragment [1].

In an attempt to assess the influence of reduction quality on the cut-out event, 82 non-cut-out patients could be matched to the 54 cut-out cases according to the variables age, fracture classification and gender. For three of the cut-out cases no equivalent patient could be found. A radiologist (DC) evaluated the quality of reduction separately for five fracture groups: AO/ASIF 31-A1, 31-A2, 31-A3, 31-B2.1 and subtrochanteric fractures.

**Figure 1:**
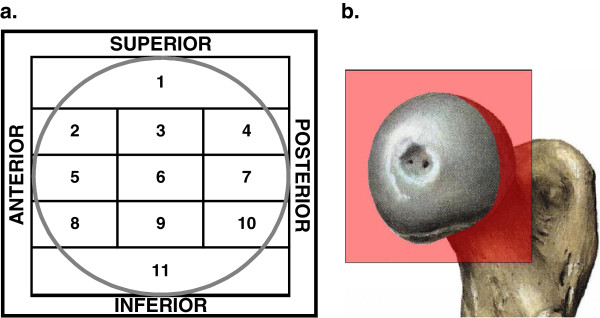
**Assessment of lag screw positioning in the femoral head. a.** the eleven-zone template of the head; **b.** the sagittal plane of the head, in which the screw position was recorded.

Lag screw position in the femoral head and nail placement in the shaft were determined from the immediate post-operative anterior-posterior and lateral radiographs. To assess lag screw position, the placement of the tip of the screw in the femoral head was considered. The position was recorded according to the modified eleven-zones-template of the femoral head. By dividing the head into four zones on the A-P view and three zones on the lateral view, the position was plotted on the sagittal plane (Figure 1). The reason for this modification, after Kyle et al. [11], was to distinguish more precisely between two locations described in the literature to be optimal: central- central and central-inferior zone as seen on the lateral and A-P view.

An additional goal was to assess the position of the lag screw by means of Tip-Apex Distance (TAD) [1]. However, the varying quality of immediate postoperative radiographic records in this study did not allow collecting a valid amount of data.

Cut-out was defined as projection of the lag screw from the femoral head by more than 1 mm [12].

The institutional review board at CTO gave ethical approval before the study was commenced. Due to the retrospective nature of the study no burden or risk was imposed on the patients.

### Statistical analysis

Results were tabulated and statistically analysed by using the IBM SPSS (version 17, SPSS Inc. Chicago, Illinois, USA). The SPSS database was uniquely designed for our patient population and the questions adapted with the help of patient notes samples before starting the study. Frequency and relative distribution were presented in tabular form for categorical variables. Comparative analysis was performed with the Chi-Square or Fisher Exact (for 2x2 tables) test for categorical variables. Before analysing continuous variables, the data sets were assessed for normality by performing the Shapiro-Wilk test. When distribution was considered to be normal, two-sided Student’s t-test was performed; otherwise the Mann–Whitney test was used (confidence level for all tests = 95%). P-value <0.05 was considered statistically significant.

## Results

71 (2.3%) cut-out complications were identified with the help of clinical and radiographic records.

The group of 71 complications was divided into primary (57 cases, 1.85%) and secondary cut-outs (14 cases, 0.45%). Cut-out was defined as being secondary if it was caused by pathological tissue (other than osteoporosis) such as avascular head necrosis, bone metastasis, osteomyelitis or as a secondary outcome to prior implant failure and not the biomechanical pattern of the osteosynthesis. The secondary cut-out cases were identified and excluded from the further analysis (Table 1).

**Table 1:** Patients with secondary cut-out excluded from the study

	**Reason for secondary cut-out**	**Age**	**Sex**	**Coexistent disease**	**Fracture pattern**	**Time between OP and complication diagnosis**	**Treatment**
1	AVN	67	M	Alcoholism	31-A3.3	5 months	THR
2	AVN	79	F		31-A3.3	8 months	Girdelstone procedure
3	AVN	54	M	Alcoholism	31-B2.1	24 months	Lost to follow-up
4	AVN	80	F	-	31-A1.2	10 months	Lost to follow-up
5	AVN	59	F	Parkinson disease	31-A1.1	6 months	THR
6	Bone metastasis	65	F	Breast cancer	Pathologic	7 months	Nail change, bone graft
7	Bone metastasis	80	F	Breast cancer	Pathologic	3 months	Lost to follow-up
8	Osteomyelitis	74	F	-	31-A1.2	5 months	THR
9	Osteomyelitis	79	F	-	31-A1.2	5 months	THR
10	Revision of cut-out on Ender nail	65	M	-	-	5 months	THR
11	Revision of fracture on triple screws after iterative fall	41	M	Epilepsy	-	1.5 months	Shorter lag screw
12	Revision of non-union	32	M	Epilepsy	-	5 months	THR
13	Revision of cut-out in Gamma Nail					12 months	THR
14	Revision of cut-out in Gamma Nail	66	M	Alcoholism	-	28 months	Lost to follow-up

### Primary cut-out complications

Cut-out was defined as being primary when the reason for this failure was of biomechanical origin, i.e. it was determined by fracture geometry before and after fracture reduction and by the lag screw positioning.

Fifty-seven patients (median age 82.6 years, 12/45 males/females) were identified. 97% of the factures were caused by low-energy trauma and two fractures by high-energy trauma. Six patients had an associated injury.

In 45 cases (79%) cut-out occurred within first 12 weeks after surgery (range 8 to 670 days). Twenty-one patients (37%) received no surgical treatment of this complication due to advanced age, major medical co-morbidities and low functional demands. Thirty-six patients underwent revision procedure. In 13 cases a total hip replacement and in four cases unipolar hip replacement were performed. The nail was exchanged in seven patients. Removal of the nail after consolidation was carried out in seven patients. Five patients, for whom the surgical treatment was planned, were lost to follow up.

### Cut-out patterns

**Figure 2:**
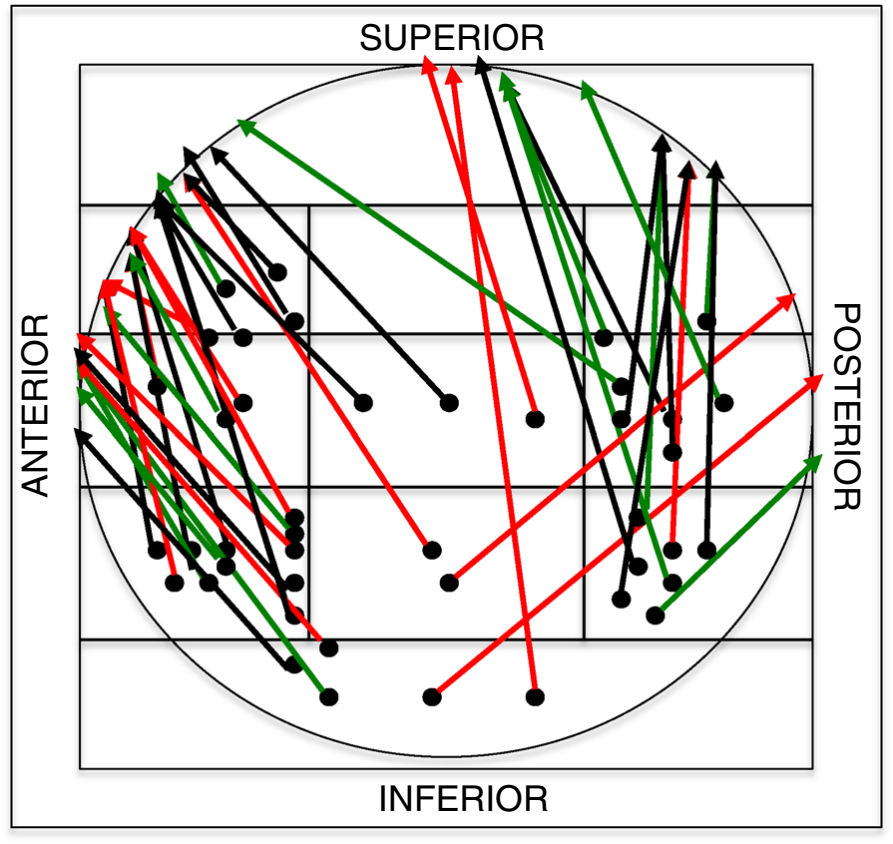
**Cut-out patterns (two-dimensional interpretation).** Primary position of the lag screw in the femoral head (points in the zone template), direction of migration and approximate penetration point of the lag screw (arrows). Red arrows: 31-B2.1 (basicervical) fractures, green arrows: 31-A3.3 fractures, black arrows : other fractures; 43 cases, central cut-out has not been considered.

A number of different configurations of cut-out were observed in relation to the primary position of the lag screw, its migration and approximate penetration point in the femoral head (Figure 2).

We observed that the majority of the lag screws migrated anteriorly-superiorly relatively to its intraoperative position. Only three lag screws migrated posteriorly (Figure 2). Central cut-out (along the lag screw axis) occurred in eight patients. In six cases the lag screw was prevented from sufficient lateral sliding. In two cases the lag screw migrated medially relative to the nail. In another six cases, the assessment of the lag screw migration was not possible because of insufficient quality of radiographs.

### Cut-out complication over time

**Figure 3:**
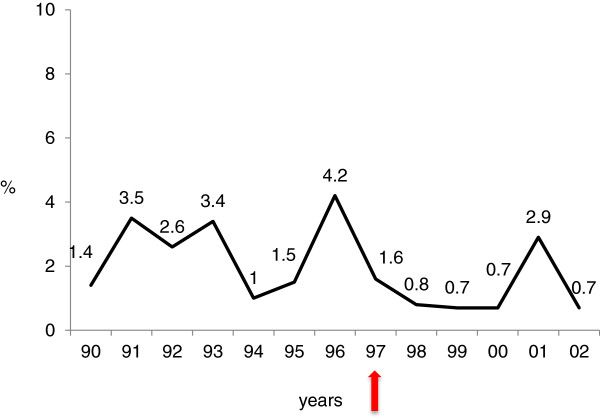
**Yearly distribution of cut-out complication.** Red arrow: introduction of the Trochaneric Gamma Nail.

Cut-out frequency varied slightly over the twelve years. After introduction of the Trochanteric Gamma Nail in 1997, the cut-out rate fell from 2.5% to 1.1% (p=0.031) (Figure 3). The distribution of the fracture types did not differ over the years for the cut-out cases.

### Analysis of factors in 57 primary cut-out complications

#### Patients’ age

**Figure 4:**
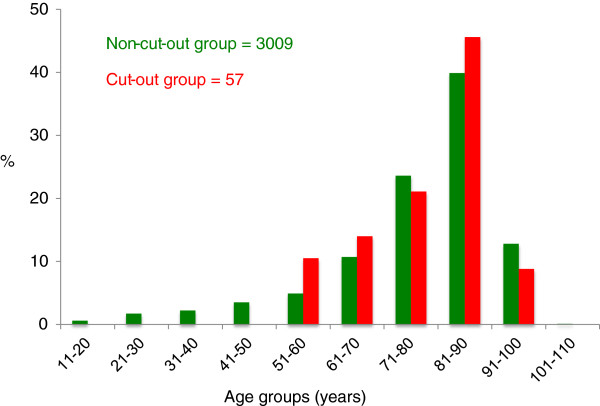
Comparison of relative frequency of patient age groups for the non-cut-out group (n= 3009), and cut-out group (n= 57).

The age distribution was similar in both groups (cut-out and non-cut-out group) with a peak between 81 and 90 years (Figure 4). No cut-out occurred in patients younger than 50 years.

#### Neck-shaft angle of the nail

Cut-out rate has been analysed in the Standard Gamma Nail group with respect to the neck-shaft angle of the nail. There were six cut-out cases among 296 nails with 125° of neck shaft angle, 37 cases among 1239 nails of 130° and one case out of 80 nails of 135°. There were no statistically significant differences between the groups.

#### Implant design: SGN versus TGN

Among 1623 SGNs cut-out occurred in 44 cases, there were 10 cut-outs out of 933 TGNs, and 3 cases among 473 LGNs. The difference between SGN and TGN was highly significant (p=0.006). LGN was not comparable with short Gamma Nails due to its use in younger patients with predominantly trochantero-diaphyseal fractures.

#### Fracture pattern

**Figure 5:**
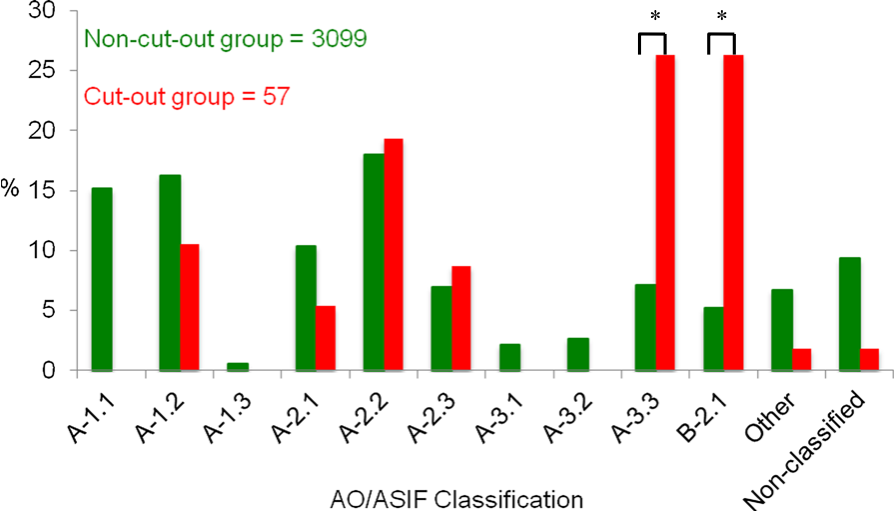
Comparison of the fracture types for proximal femur (AO 31) between non-cut-out and cut-out group. * statistically significant overrepresentation (p<0.001).

There was a statistically significant over-representation of complex unstable 31-A3.3 fractures (26.3%) and basicervical 31-B2.1 fractures (26.3%) in the cut-out group (p<0.001) (Figure 5). The fractures labelled as “other” were not taken into consideration, since these were combined trochantero-diaphyseal femoral fractures occurring mainly in younger patients (mean age 64.9 years) treated with LGN.

#### Fracture reduction

In 44 fractures in the cut-out group, the fracture reduction was not anatomical. There was no statistically significant difference (p=0.55) in reduction quality between the cut-out and the matched group. However, there was a slight overrepresentation of non-anatomically reduced fractures the basicervical group (31-B2.1) (p=0.089).

#### Lag screw positioning in the femoral head

**Figure 6:**
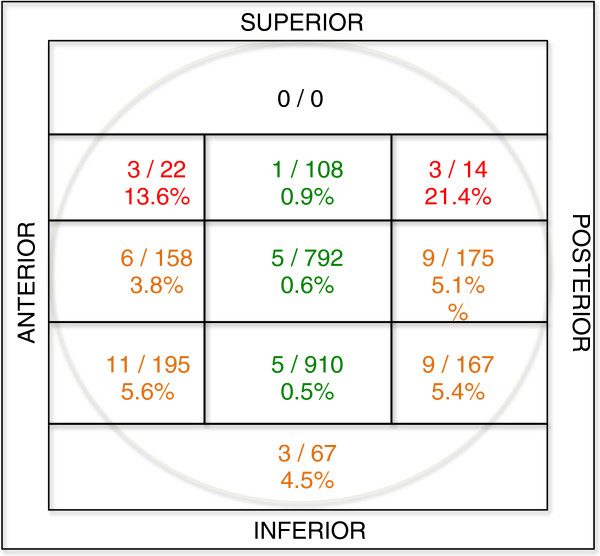
**Frequency of lag screw cut-out in relation to the position in the femoral head.** Total number of screws in each zone is represented by the numerator (n = 2610), and the number of screws that cut-out in each zone is represented by the denominator (n = 55).

Lag screws were found to have been placed in all possible locations within the femoral head, but the very cranial one, as evaluated according to a modified eleven-zones template used by Kyle [11] (Figure 6).

### Interrelations between critical factors in cut-out complication

**Figure 7:**
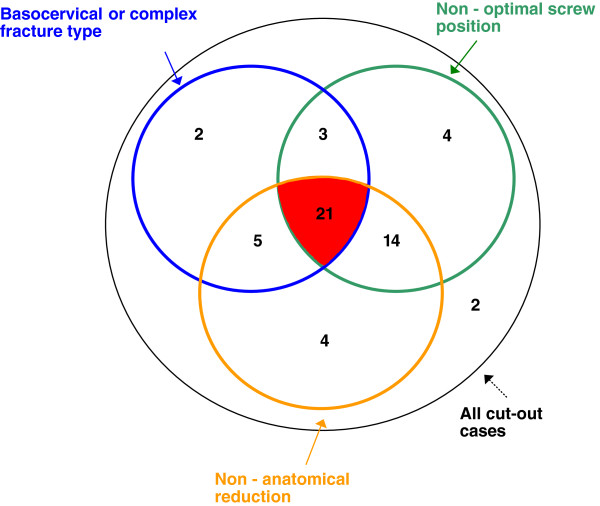
**Venn diagram: interrelations between critical cut-out factors.** The figures represent number of cases in each category.

An unstable and complex fracture pattern, non-anatomical reduction and non-optimal positioning of the lag screw were found to be critical factors contributing to the cut-out complication. The interrelations between these factors are presented in Figure 7. All cut-out cases but two had at least one of the mentioned features. 31 patients presented a typical fracture type. These were basicervical (AO 31-B2.1) or trochanteric with comminute “pantrochanteric” fracture pattern (AO 31-A3.3). In 44 cases reduction of the fracture was assessed as non-anatomical. The lag screw was malpositioned (outside of the zones 3,6,9, Figure 6) in 42 patients. The combination of all three factors or the combination of non-anatomical reduction and non-optimal lag screw positioning was found strongly predictive for the cut-out. There were two patients without any contributing factors: a 78-year-old woman and an 80-year-old man. These patients had fractures classified respectively as AO/ASIF 31-A1.2 and 31-A2.1 with corresponding cut-outs occurring at 7 and at 8 months after surgery. Whilst they were assessed to have had stable fractures primarily, they both had non-unions at the base of the neck at the time of cut-out failure, suggesting that they had been incorrectly classified initially.

## Discussion

The prevalence of cut-out failure in this large consecutive series of 3066 proximal femoral fractures was 1.85% (57 patients). Four factors were found to contribute to this important complication: non-anatomical reduction, non-optimal screw position, complex fracture pattern and implant design. The combination of the first three of them further increased the likelihood of cut-out.

The newer implant designs correlated with significantly less cut-out complications. Other factors like patients’ age and CCD-nail angle did not affect the outcome. The two-dimensional analysis of the cut-out pattern showed predominantly anterior-superior migration of the lag screw out of the femoral head.

### Cut-out pattern

In previous reports, cut-out has been evaluated on two-dimensional radiographs, which show varus collapse of the femoral head and superior cut-out of the lag screw [1,13]. However, in vivo telemetry study of hip implants shows surprisingly large rotational moments acting on the femoral head during gait caused by the A-P force [14,15]. The authors suggest that these forces should be taken into consideration in the implant design process. Further, recent biomechanical studies direct attention to a multiplanar mechanism of cut-out, due to combined axial loads and rotational moments acting during walking gait [16,17]. Under these conditions the mechanism of cut-out exhibits combined varus collapse and backward rotation provided that the lag screw have been placed in the central position in the femoral head [17]. In the present study, the cranial migration of the lag screw and varus collapse of the femoral head were accompanied by predominantly anterior movement. This observation supports the findings in the studies mentioned above. Central cut-out (along the lag screw axis) was associated with the failure of the sliding mechanism and simultaneous instability of the fracture. This can be explained by the inaccurate use of the set screw either by over-tightening preventing the lag screw from sliding or by failure to engage the set screw in the lag screw notch, thus allowing uncontrolled lag screw rotation and central migration toward acetabulum. This cut-out pattern has already been described in the literature [18].

### Lag screw positioning in the femoral head

The optimal position of the lag screw has been widely discussed in the literature, particularly the aspect of central [11,19,20] or inferior [21-25] placement of the screw in the femoral head as seen on the A-P view .

The importance of a central placement of the screw on the lateral radiograph has been emphasised in the literature [3,12,26]. Central placement of the screw reduces the risk of rotation of the femoral head and neck around the screw (small torsional moment) that can occur with eccentric placement [27,28].

The present study emphasizes the importance of placing the lag screw in the centre of the femoral head on the lateral radiograph. However, it was not possible to define a single optimal zone (inferior, central or even slightly superior) on the A-P view. This finding correlates precisely with the statement of Davis [3] that the cut-out rate was not significantly affected by either a superior or an inferior placement as seen on the A-P view if the implants were centrally positioned on the lateral radiograph. This indirectly supports the concept of tip-apex distance [1]: the placement of the lag screw close to the apex of the femoral head on A-P view with the central placement on the lateral view is essential for the outcome.

### Fracture reduction

The majority of the cut-out cases displayed non-anatomical reduction (44/57) and a number of studies show a statistically significant relationship between non-anatomical reduction and cut-out complication [29-31]. However, in the comparison with the matching controls in our study, no clear difference could be shown perhaps due to limited sample size. Nevertheless, the basicervical fractures with cut-out had a slight overrepresentation of non-anatomical reduction (p=0.089). A possible explanation for the poor reduction could be the complexity of these fractures (AO/ASIF 31-A3.3).

### Fracture pattern

In the present study, the cut-out rate for AO/ASIF 31- A3.3 fracture was 6.5%. The complication rate for basicervical fracture was even higher - 9%, while overall cut-out rate was 1.0% (AO/ASIF 31- A3.3 and B2.1 excluded).

The fracture type (its complexity and concomitant stability) has been recognised as an important factor contributing to the osteosynthesis failure [3,32,33]. The treatment of fractures that have two fragments is usually associated with fewer complications [30,34]. The increased rate of mechanical failure in complex inter-trochanteric fractures with subtrochanteric or cervical extension has been described in the literature. Haidukiewych [32] reported on high cut-out rate of 12.7% in 47 reverse obliquity fractures (AO/ASIF 31-A3.1 and A3.3) regardless of the type of internal fixation devices. The author also observed worse results for fractures with poor reduction or poor implant position in the femoral head. The treatment of this type of fracture with a Gamma Nail can also result in a high risk of cut-out regardless of the lag screw location [26,35].

Only few reports focus on basicervical fractures as a separate entity [36,37], these demonstrate a high incidence of local complications including cut-out leading to re-operations.

### Implant design

Clinical studies have consistently failed to find statistically significant differences between implant designs with regard to lag screw cut-out [38-40].

In the present study, cut-out rates decreased significantly when the SGN was replaced by TGN (2.7% vs. 1.1%, p= 0.006). This might have been caused by the improved design of the second-generation nail: reduced valgus bend of 4°, reduced length of 180 mm and only one distal locking hole (the lag screw itself was not modified). These design changes eliminated the concept of 3-point contact of the nail in the femoral shaft [4] and subsequently could have optimised the positioning of the lag screw in the femoral head. The influence of the improved implant design on the decreasing complication rate in hip fractures has been recently shown by Bhandari et al. [41]. The meta-analysis suggests that newer intramedullary nail designs have reduced the risk of femoral shaft fracture.

### Neck-shaft angle

The influence of the neck-shaft angle of an implant on cut-out has been controversial. The clinical study by Kukla [4] showed a significant increase in cut-outs in higher angle implants. On the contrary, biomechanical studies show that higher angles, in order to enhance sliding of the screw and fracture site impaction, result in less cut-out [42-45]. In the present study, the analysis of the CCD-nail angle did not reveal any difference for this parameter.

### Age

The present study supports the finding that age does not determine the cut-out rate [3]. There was no statistically significant age difference between the non-cut-out and the cut-out group, although this complication did not occur in patients younger than 50 years. On the other hand, there are clinical studies where increasing age of the patient is found to be predictive of implant failure [1,29,46].

### Bone quality

The opinion that poor bone quality increases the mechanical failure rate of an osteosynthesis is widely represented [30,44,47]. However, some authors have shown with help of Singh index or quantitative computed tomography (QCT) that cut-out is not influenced by this factor [3,26,46]. In the present study the bone quality was not assessed because of lack of reliable methods, however the patients with cut-out complication were considered to have osteoporotic bone based on age, gender and the presence of the low-energy fracture [13,48,49]. We were unable to identify the osteoporosis as a contributing factor and all but two cut-outs could be explained by the presence of other factors (Figure 7).

### Limitations of the study

The review of large consecutive series of proximal femoral fractures enables statistically supported statements. On the other hand, the retrospective nature leads to some limitations in interpretation caused by loss to follow-up, non-standardised methods of data collection or inconsistent quality of radiographs. However, the prospective record of all Gamma Nails kept at the hospital since the introduction of this implant in the late 80’s allowed demographic and technical intra-operative data to be completed.

We therefore believe that the results and particularly the cut-out rate in the present study are reliable. This complication is associated with an important pain and reduction of function, forcing the patient to look for help at the CTO.

## Conclusions

This study has identified three variables associated with high risk of cut-out: unstable fracture type (basicervical and complex fractures), non-anatomical reduction and non-optimal lag screw positioning. These factors are closely interdependent since complex fractures may be difficult to reduce which in turn leads to difficulties in achieving the correct positioning of the implant. These observations underscore the importance of correct operative technique. Therefore, surgeons confronted with proximal femoral fractures should carefully scrutinize preoperative radiographs to assess the primary fracture geometry and classification. It is important to achieve anatomical reduction in order to place the lag screw in the optimal position and to avoid the complication of cut-out, the only two factors that can be controlled by the surgeon.

New approaches should be made to improve reduction quality and implant positioning to ensure better results in complex fractures such as improved fracture imagining.

## Competing interests

CB is an employee of Stryker Osteosynthesis GmbH, Germany, GT is a consultant and AJB was an employee of Stryker Osteosynthesis GmbH during the data collection period.

## Authors’ contributions

AJB carried out the data collection, analysis and participated in manuscript writing. CB participated in study design performed statistical data analysis. DC evaluated the fracture reduction for the cut-out and matched groups. AJ and CE participated in data analysis and manuscript writing. AS and GT participated in study design and coordination and help to draft the manuscript. All authors read and approved the final manuscript.

## Pre-publication history

The pre-publication history for this paper can be accessed here:

http://www.biomedcentral.com/1471-2474/14/1/prepub

## References

[B1] Baumgaertner MR Curtin SL Lindskog DM Keggi JM The value of the tip-apex distance in predicting failure of fixation of peritrochanteric fractures of the hip J Bone Joint Surg Am 1995 77 7 1058 1064 7608228 10.2106/00004623-199507000-00012

[B2] Nordin S Zulkifli O Faisham WI Mechanical failure of Dynamic Hip Screw (DHS) fixation in intertrochanteric fracture of the femur Med J Malaysia 2001 56 Suppl D 12 17 14569760

[B3] Davis TR Sher JL Horsman A Simpson M Porter BB Checketts RG Intertrochanteric femoral fractures. Mechanical failure after internal fixation J Bone Joint Surg Br 1990 72 1 26 31 2298790 10.1302/0301-620X.72B1.2298790

[B4] Kukla C Heinz T Gaebler C Heinze G Vecsei V The standard Gamma nail: a critical analysis of 1,000 cases J Trauma 2001 51 1 77 83 10.1097/00005373-200107000-00012 11468471

[B5] Utrilla AL Reig JS Munoz FM Tufanisco CB Trochanteric gamma nail and compression hip screw for trochanteric fractures: a randomized, prospective, comparative study in 210 elderly patients with a new design of the gamma nail J Orthop Trauma 2005 19 4 229 233 10.1097/01.bot.0000151819.95075.ad 15795570

[B6] Wolfgang GL Bryant MH O'Neill JP Treatment of intertrochanteric fracture of the femur using sliding screw plate fixation Clin Orthop 1982 163 148 158 7067245

[B7] Simpson AH Varty K Dodd CA Sliding hip screws: modes of failure Injury 1989 20 4 227 231 10.1016/0020-1383(89)90120-4 2592101

[B8] Wu CC Shih CH Chen WJ Tai CL Treatment of cutout of a lag screw of a dynamic hip screw in an intertrochanteric fracture Arch Orthop Trauma Surg 1998 117 4–5 193 196 9581243 10.1007/s004020050228

[B9] Bojan AJ Beimel C Speitling A Taglang G Ekholm C Jonsson A 3066 consecutive Gamma Nails. 12 years experience at a single centre BMC Musculoskelet Disord 2010 11 133 10.1186/1471-2474-11-133 20579384 PMC2906434

[B10] Garden RS Low-angle fixation in fractures of the femoral neck Journal Joint Bone Surg Br 1961 43-B 4 647 663

[B11] Kyle RF Gustilo RB Premer RF Analysis of six hundred and twenty-two intertrochanteric hip fractures J Bone Joint Surg Am 1979 61 2 216 221 422605

[B12] Parker MJ Cutting-out of the dynamic hip screw related to its position J Bone Joint Surg Br 1992 74 4 625 1624529 10.1302/0301-620X.74B4.1624529

[B13] Baumgaertner MR Solberg BD Awareness of tip-apex distance reduces failure of fixation of trochanteric fractures of the hip J Bone Joint Surg Br 1997 79 6 969 971 10.1302/0301-620X.79B6.7949 9393914

[B14] Brown RH Burstein AH Frankel VH Telemetering in vivo loads from nail plate implants J Biomech 1982 15 11 815 823 10.1016/0021-9290(82)90046-X 7161283

[B15] Bergmann G Graichen F Rohlmann A Hip joint loading during walking and running, measured in two patients J Biomech 1993 26 8 969 990 10.1016/0021-9290(93)90058-M 8349721

[B16] Sommers MB Roth C Hall H Kam BC Ehmke LW Krieg JC Madey SM Bottlang M A laboratory model to evaluate cutout resistance of implants for pertrochanteric fracture fixation J Orthop Trauma 2004 18 6 361 368 10.1097/00005131-200407000-00006 15213501

[B17] Ehmke LW Fitzpatrick DC Krieg JC Madey SM Bottlang M Lag screws for hip fracture fixation: evaluation of migration resistance under simulated walking J Orthop Res 2005 23 6 1329 1335 15994054 10.1016/j.orthres.2005.05.002.1100230614

[B18] Heinz T Vecsei V [Complications and errors in use of the gamma nail. Causes and prevention] Chirurg 1994 65 11 943 952 7821075

[B19] Mulholland R Gunn DR Sliding screw plate fixation of intertrochanteric femoral fractures J Trauma 1972 12 7 581 591 10.1097/00005373-197207000-00006 5044317

[B20] Davis J Harris MB Duval M D'Ambrosia R Pertrochanteric fractures treated with the Gamma nail: technique and report of early results Orthopedics 1991 14 9 939 942 1946058 10.3928/0147-7447-19910901-05

[B21] Wu CC Shih CH Biomechanical analysis of the dynamic hip screw in the treatment of intertrochanteric fractures Arch Orthop Trauma Surg 1991 110 6 307 310 10.1007/BF00443464 1747312

[B22] Wu CC Shih CH Lee MY Tai CL Biomechanical analysis of location of lag screw of a dynamic hip screw in treatment of unstable intertrochanteric fracture J Trauma 1996 41 4 699 702 10.1097/00005373-199610000-00017 8858031

[B23] Mainds CC Newman RJ Implant failures in patients with proximal fractures of the femur treated with a sliding screw device Injury 1989 20 2 98 100 10.1016/0020-1383(89)90151-4 2592088

[B24] Levi N Ingles A Jr Klyver H Iversen BF Fracture of the femoral neck: optimal screw position and bone density determined by computer tomography Injury 1996 27 4 287 289 10.1016/0020-1383(95)00207-3 8762791

[B25] Wagner S Ruter A [Per- and subtrochanteric femur fractures] Unfallchirurg 1999 102 3 206 222 10.1007/s001130050394 10232037

[B26] Kawaguchi S Sawada K Nabeta Y Cutting-out of the lag screw after internal fixation with the Asiatic gamma nail Injury 1998 29 1 47 53 10.1016/S0020-1383(97)00158-7 9659482

[B27] Den Hartog BD Bartal E Cooke F Treatment of the unstable intertrochanteric fracture. Effect of the placement of the screw, its angle of insertion, and osteotomy J Bone Joint Surg Am 1991 73 5 726 733 2045397

[B28] Lenich A Bachmeier S Prantl L Nerlich M Hammer J Mayr E Al-Munajjed AA Fuchtmeier B Is the rotation of the femoral head a potential initiation for cutting out? A theoretical and experimental approach BMC Musculoskelet Disord 2011 12 79 10.1186/1471-2474-12-79 21513536 PMC3108935

[B29] Hsueh KK Fang CK Chen CM Su YP Wu HF Chiu FY Risk factors in cutout of sliding hip screw in intertrochanteric fractures: an evaluation of 937 patients Int Orthop 2010 34 8 1273 1276 10.1007/s00264-009-0866-2 19784649 PMC2989068

[B30] Larsson S Friberg S Hansson LI Trochanteric fractures. Influence of reduction and implant position on impaction and complications Clin Orthop 1990 259 130 139 2208847

[B31] Pervez H Parker MJ Vowler S Prediction of fixation failure after sliding hip screw fixation Injury 2004 35 10 994 998 10.1016/j.injury.2003.10.028 15351665

[B32] Haidukewych GJ Israel TA Berry DJ Reverse obliquity fractures of the intertrochanteric region of the femur J Bone Joint Surg Am 2001 83-A 5 643 650 11379732 10.2106/00004623-200105000-00001

[B33] Jensen JS Sonne-Holm S Tondevold E Unstable trochanteric fractures. A comparative analysis of four methods of internal fixation Acta Orthop Scand 1980 51 6 949 962 10.3109/17453678008990900 7211302

[B34] Lyddon DW Jr The prevention of complications with the Gamma Locking Nail Am J Orthop 1996 25 5 357 363 8727086

[B35] Kyle RF Ellis TJ Templeman DC Surgical treatment of intertrochanteric hip fractures with associated femoral neck fractures using a sliding hip screw J Orthop Trauma 2005 19 1 1 4 10.1097/00005131-200501000-00001 15668576

[B36] Saarenpaa I Partanen J Jalovaara P Basicervical fracture-a rare type of hip fracture Arch Orthop Trauma Surg 2002 122 2 69 72 10.1007/s004020100306 11880905

[B37] Su BW Heyworth BE Protopsaltis TS Lipton CB Sinicropi SM Chapman CB Kuremsky MA Rosenwasser MP Basicervical versus intertrochanteric fractures: an analysis of radiographic and functional outcomes Orthopedics 2006 29 10 919 925 17061418 10.3928/01477447-20061001-04

[B38] Haynes RC Poll RG Miles AW Weston RB Failure of femoral head fixation: a cadaveric analysis of lag screw cut-out with the gamma locking nail and AO dynamic hip screw Injury 1997 28 5–6 337 341 9764228 10.1016/s0020-1383(97)00035-1

[B39] Audige L Hanson B Swiontkowski MF Implant-related complications in the treatment of unstable intertrochanteric fractures: meta-analysis of dynamic screw-plate versus dynamic screw-intramedullary nail devices Int Orthop 2003 27 4 197 203 10.1007/s00264-003-0457-6 12734684 PMC3458474

[B40] Fritz T Hiersemann K Krieglstein C Friedl W Prospective randomized comparison of gliding nail and gamma nail in the therapy of trochanteric fractures Arch Orthop Trauma Surg 1999 119 1–2 1 6 10076936 10.1007/s004020050345

[B41] Bhandari M Schemitsch E Jonsson A Zlowodzki M Haidukewych GJ Gamma nails revisited: gamma nails versus compression hip screws in the management of intertrochanteric fractures of the hip: a meta-analysis J Orthop Trauma 2009 23 6 460 464 10.1097/BOT.0b013e318162f67f 19550235

[B42] Loch DA Kyle RF Bechtold JE Kane M Anderson K Sherman RE Forces required to initiate sliding in second-generation intramedullary nails J Bone Joint Surg Am 1998 80 11 1626 1631 9840631 10.2106/00004623-199811000-00009

[B43] Meislin RJ Zuckerman JD Kummer FJ Frankel VH A biomechanical analysis of the sliding hip screw: the question of plate angle J Orthop Trauma 1990 4 2 130 136 10.1097/00005131-199004020-00005 2358926

[B44] Flores LA Harrington IJ Heller M The stability of intertrochanteric fractures treated with a sliding screw-plate J Bone Joint Surg Br 1990 72 1 37 40 2298792 10.1302/0301-620X.72B1.2298792

[B45] Kyle RF Wright TM Burstein AH Biomechanical analysis of the sliding characteristics of compression hip screws J Bone Joint Surg Am 1980 62 8 1308 1314 7440609

[B46] Andress HJ Forkel H Grubwinkler M Landes J Piltz S Hertlein H Lob G [Treatment of per- and subtrochanteric femoral fractures by gamma nails and modular hip prostheses. Differential indications and results] Unfallchirurg 2000 103 6 444 451 10.1007/s001130050564 10925646

[B47] Laros GS Intertrochanteric fractures. The role of complications of fixation Arch Surg 1975 110 1 37 40 10.1001/archsurg.1975.01360070037007 1115608

[B48] Kim WY Han CH Park JI Kim JY Failure of intertrochanteric fracture fixation with a dynamic hip screw in relation to pre-operative fracture stability and osteoporosis Int Orthop 2001 25 6 360 362 10.1007/s002640100287 11820441 PMC3620783

[B49] Kanis JA Johnell O Oden A Jonsson B De Laet C Dawson A Risk of hip fracture according to the World Health Organization criteria for osteopenia and osteoporosis Bone 2000 27 5 585 590 10.1016/S8756-3282(00)00381-1 11062343

